# Reply to Foster, K.R.; Balzano, Q. Comment on “Redmayne, M.; Maisch, D.R. ICNIRP Guidelines’ Exposure Assessment Method for 5G Millimetre Wave Radiation May Trigger Adverse Effects. *Int. J. Environ. Res. Public Health* 2023, *20*, 5267”

**DOI:** 10.3390/ijerph20227031

**Published:** 2023-11-07

**Authors:** Mary Redmayne, Donald R. Maisch

**Affiliations:** 1 School of Geography, Environment and Earth Sciences, Victoria University of Wellington, Kelburn Parade, Wellington 6012, New Zealand; 2 Oceania Radiofrequency Scientific Advisory Association Inc. (ORSAA), Brisbane, QLD 4020, Australia; dmaisch@emfacts.com; 3 The Australasian College of Nutritional and Environmental Medicine (ACNEM), Melbourne, VIC 3205, Australia

We would like to thank Foster and Balzano for their interest in our paper [1]. We note the lengthy analysis of why they believe the earlier of the Albenese papers that we discussed is not relevant. 

However, their comment was focused on just a couple of our points, omitting the mention of an important underlying one. 

Simply put, the main thrust of the paper is that research indicates that Specific Absorption Rate readings can be considerably higher than expected when surface measures are within the ICNIRP Guidelines’ allowances. Adhering to these guidelines, which only require surface measurements of mmW exposure for localized areas, may result in heat injury. We believe this applies regardless of whether Brillouin pulses are invoked by 5G under beam-formed circumstances, which we discussed. In fact, as we pointed out, structures nearest the skin’s surface will be preferentially heated from mmW frequency use for beamforming. 

We gratefully acknowledge Foster and Balzano in pointing out that an “idealized waveform” as calculated by them is necessary for Brillouin precursors to “cover the full spectrum of frequencies for −∞ to +∞”. We reported that “the likelihood of producing Brillouin precursors increases …with a GHz bandwidth of more the 500 MHz”. Foster and Balzano imply that this will not be available as the 5G NR “signal must remain within the frequency band assigned to the carrier”. True, it must, but this bandwidth is likely to be available. Nokia reports that “GSMA recommends that regulators and government agencies that control 5G spectrum allocation make … about 1 GHz per operator available in millimeter wave bands” [2].

Regarding the "other comment", far from being "broad" or "superficial", our critique focused on one specific aspect of the updated ICNIRP guidelines, and our selection of the supporting literature focused on that.

It was relevant to quote Neufeld and Kuster’s calculation, which indicates the possibility of “intense surface heating” [3]. We now refer the reader to the discussion that followed, the thrust of which was a challenge from Foster [4] (one of the authors of the comment to which we are replying) and Neufeld and Kuster’s clear response [5]. We feel that that response adequately removed the need to air that discussion or withdraw our reference to that paper. Further, we agree with Neufeld and Kuster’s concern that, “in view of standards and rapid technological changes, that a standard should be intrinsically safe and consistent, rather than relying on implicit assumptions about current and future technological limitations” [5].

Our reference to "homogeneous models" of dermal tissue was intended to highlight the problem of under-estimation that such models can produce, as revealed by the cited stratified model assessed by Christ et al. [6]. Foster and Balzano point out that we have overlooked some multi-layer models cited in the ICNIRP 2020 Guidelines. Two of the three papers they provide as examples include authors Akimasa Hirata and Soichi Watanabi, who were part of the ICNIRP Commission at the time the 2020 Guidelines were developed, so we do not regard these as suitably independent models to cite when critiquing ICNIRP’s Guidelines. 

We take this opportunity to add the following italicized words to the first paragraph in question (from the third page of our paper): “It needs to be borne in mind that *several* skin modelling studies treat the skin as homogeneous dermal tissue although it is a complex organ interacting with the environment …. The induced temperature increase *in such models* can be underestimated by more than a factor of three” [7]. 

The other suggested paper (Ziskin et al. [8]) includes the authors of the comment under discussion, and it could have been well cited in our paper which is under discussion here. Three of its authors, Foster, Ziskin, and Balzano, have also published a useful review of the thermal response of human skin to microwave energy (30–300 GHz) [9]. In that review, the authors make several interesting points that are pertinent to this discussion:

- Their literature search found very few studies on heating in human tissue at 5G-relevant frequencies, and those studies’ limitations were “striking” (p. 539).
- Using these data, they developed a thermal model of “extreme simplicity [with] a number of drastic approximations” (p. 529) for estimating mmW exposure levels for small areas of skin.
- They recommended it as suitable “to aid in the development and evaluation of RF safety limits at frequencies above 3 GHz and for millimeter waves” (p. 528), although there were very few data available on small skin-area exposure longer than a few minutes. We note that the paper, and presumably its model, was indeed used in the development of the ICNIRP 2020 Guidelines [10].
- They acknowledge that the Pennes’ bioheat equation on which they base it “does not account for short-range variations in temperature in the immediate vicinity of thermally significant blood vessels” (p. 536).
- There are “rather few” thermal models, including theirs, that have been “validated using data that are independent of those used to develop the models… Consequently their generalizability is uncertain” (p. 539).
- IEEE C95.1-2005 has critiqued some important limitations of various models for testing thermal compliance models, concluding, “Until these limitations can be resolved, thermal models are useful but in and of themselves are not sufficient for a safety standard development” (p. 538).
- In small, local irradiated areas, “the temperature increase at the skin surface is chiefly limited by conduction of heat into deeper tissue layers” [9] (p. 528). Yet, that deeper heating is not assessed by ICNIRP 2020 Guidelines for mmW exposures.

We agree that many research questions remain, especially with respect to the best method of assessing near-field exposure from handsets transmitting at millimeter wave frequencies. Near-field measurements have always presented challenges, and these challenges are greater for the more complex 5G mm wave exposure. This is very pertinent because so many people carry handsets in their hands or tight-fitting clothing. Until near-field assessments are reliable, we maintain that a responsible approach would be for manufacturers and regulators to advise users not to carry the device in these ways. 

We reiterate our previous comments on the urgent need to re-evaluate the ICNIRP guidelines’ basic approach and assumptions. 
